# Supplementary material for: ALKBH5 Protects Against Hepatic Ischemia–Reperfusion Injury by Regulating YTHDF1-Mediated YAP Expression
Source: Int J Mol Sci. 2024 Oct 27;25(21):11537. doi: 10.3390/ijms252111537 (PMC11546256; doi:10.3390/ijms252111537)
Supplement: Supplementary file 1 [file ijms-25-11537-s001.zip › ijms-3163080-supplementary.pdf]

Supplementary data

ALKBH5 Protects Against Hepatic Ischemia-Reperfusion Injury by Regulating YTHDF1-mediated YAP Expression

Pi-Xiao Wang[a, 1, *], Ling Zhu [a, 1], Mei Xiang[b, 1], Rixin Zhang[a], Xiaolin Zheng[a], Zhi Zheng[a], Kai Li[a, *]

[a] Department of Hepatobiliary and Pancreatic Surgery, The Central Hospital of Wuhan, Tongji Medical College, Huazhong University of Science and Technology, Wuhan, 430014, China

[b] Department of Cardiology, The Central Hospital of Wuhan, Tongji Medical College, Huazhong University of Science and Technology, Wuhan, 430014, China

[1] **Pi-Xiao Wang**, **Ling Zhu** and **Mei Xiang** contributed equally to the study and are co-first authors.

* **Corresponding authors'** *e-mail:* wangpx@whu.edu.cn *for* **Pi-Xiao Wang** or 2004515300093@alumni.hust.edu.cn *for* **Kai Li**.

Supplementary Figure

Figure S1. qPCR analysis confirmed the knockdown efficiency of the indicated siRNA Plasmids. **A** mRNA expression levels of ALKBH5 in L02 cells transfected with three different ALKBH5 siRNA or negative control siRNA (si-NC). **B** mRNA expression levels of YTHDF1 in L02 cells transfected with three different YTHDF1 siRNA or si-NC. **C** mRNA expression levels of YAP in L02 cells transfected with three different YAP siRNA or si-NC. *$p < 0.05$ compared to the Si-NC control group.

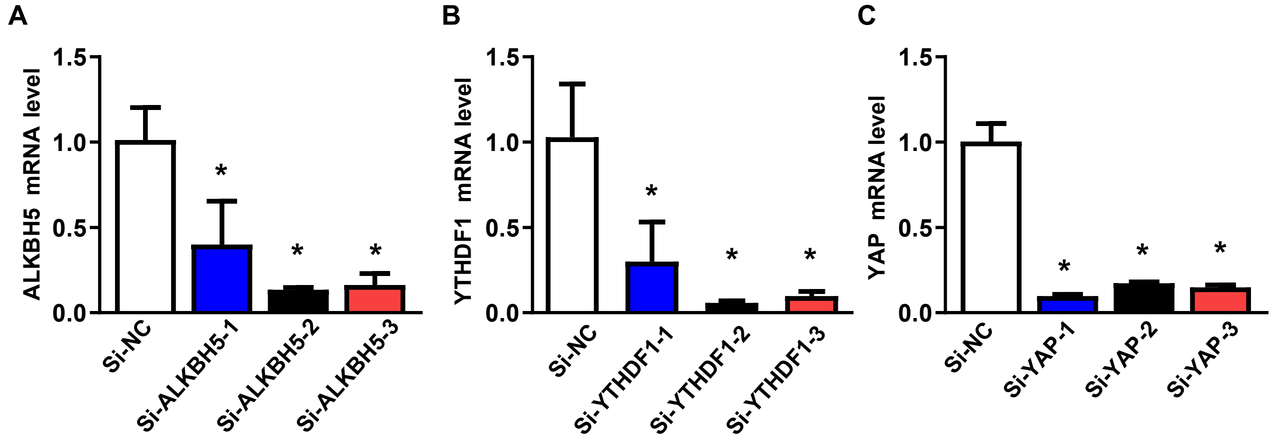

Supplementary Tables

Supplementary Table S1. Primers for real-time PCR detection.

Gene	Forward 5'-3'	Reverse 5'-3'
YAP	GTTGGGAGCTGTTTCTCCTG	GCCATGTTGTTGTCTGATCG
MYC	TTCGGGTAGTGGAAAACCAG	CAGCAGCTCGAATTTCTTCC
SPP1	TGAAACGAGTCAGCTGGATG	TGAAATTCATGGCTGTGGAA
Notch1	ACTGTGAGGACCTGGTGG	TTGTAGGTGTTGGGGAGGTC
AXIN1	TGAAGTGGGCTGAGTCACTG	CTTTCGGTAGATGGCTCTCG
β-Catenin	GGAGCCCTTCACATCCTAGC	TGCAGCTTCCTTGTCCTGAG
BIRC5	GAACTGGCCCTTCTTGGAG	TCCGCAGTTTCCTCAAATTC
YTHDF1	ACCTCACCACCTACGGACAG	GGGTCTGCTGACCTTGAGAC
ALBKH5	CGCAAGGCAGACCCTGAT	CGGTTCTCTTCCTTGTCCATCT
Ki-67	AATTCAGACTCCATGTGCCTGAG	CTTGACACACACACATTGTCCTCAGC
Bax	TGCAGAGGATGATTGCTGAC	GATCAGCTCGGGCACTTTAG
PCNA	GAAGCACCAAACCAGGAGAA	TCACTCCGTCTTTTGCACAG
Cytochrome c	CCAGCTACCATGTCCCAGAT	TATGCCAGCTTCCGACTCTT
β-Actin	TGGACTTCGAGCAAGAGATG	GAAGGAAGGCTGGAAGAGTG

Supplementary Table S2. Antibodies for immunoblot analyses.

Antibody	Cat No.	Manufacturer	Dilution
ALKBH5	16837-1-AP	Proteintech	1:1000
GAPDH	60004-1-Ig	Proteintech	1:1000
β-Actin	ab213262	Abcam	1:10000
PCNA	CST	#13110	1:2000
Cyclin D1	ab16663	Abcam	1:1000
Bcl-2	ab194583	Abcam	1:500
BAX	CST	#2772	1:1000
YAP	A1002	ABclonal	1:2000
p-YAP (S127)	ab76252	Abcam	1:2000
Smad3	Santa	sc-101154	1:1000
Tgf-β1	ab215715	Abcam	1:2000
BIRC5	10508-1-AP	Proteintech	1:1000
SPP1	22952-1-AP	Proteintech	1:1000
MYC	67447-1-Ig	Proteintech	1:5000
β-catenin	66379-1-Ig	Proteintech	1:1000
Axin1	68093-1-Ig	Proteintech	1:1000
Notch1	A22674	ABclonal	1:1000
Tubulin	11224-1-AP	Proteintech	1/3000
Histone-3	A13824	ABclonal	1/500
YTHDF1	17479-1-AP	Proteintech	1:1000
Ki67	27309-1-AP	Proteintech	1:1000
PCNA	24036-1-AP	Proteintech	1:2000
cleaved Caspase3	19677-1-AP	Proteintech	1:500
Cytochrome c	10993-1-AP	Proteintech	1:1000
BAX	50599-2-lg	Proteintech	1:2000

Supplementary Table S3. Primers for plasmid construction.

Primer name	Primer sequence	
	**Sense (5'---3')**	**Anti-Sense (5'---3')**
hALKBH5 siRNA-1	GCUGCAAGUUCCAGUUCAAGCTT	GCUUGAACUGGAACUUGCAGCTT
hALKBH5 siRNA-2	GCGCCGUCAUCAACGACUACCTT	GGUAGUCGUUGAUGACGGCGCTT
hALKBH5 siRNA-3	CCUCAGGAAGACAAGAUUAGATT	UCUAAUCUUGUCUUCCUGAGGTT
hYAP siRNA-1	GGUCAGAGAUACUUCUUAAAUTT	AUUUAAGAAGUAUCUCUGACCTT
hYAP siRNA-2	GGAGAAAUUUACUAUAUAAACTT	GUUUAUAUAGUAAAUUUCUCCTT
hYAP siRNA-3	GGUGAUACUAUCAACCAAAGCTT	GCUUUGGUUGAUAGUAUCACCTT
hYTHDF1 siRNA-1	GGAGAAUAACGACAACAAACCTT	GGUUUGUUGUCGUUAUUCUCCTT
hYTHDF1 siRNA-2	GGACAGUCAAAUCAGAGUAACTT	GUUACUCUGAUUUGACUGUCCTT
hYTHDF1 siRNA-3	GGCGUGUGUUCAUCAUCAAGATT	UCUUGAUGAUGAACACACGCCTT
Negative control siRNA	UUCUCCGAACGUGUCACGUTT	ACGUGACACGUUCGGAGAATT
hALKBH5 overexpression	CCCAAGCTGGCTAGCGTTTAAACTTAAGCTTGCCACCATGGCGGCCGCCAGCGGCTACACGGACCTGCG	GCCGCCACTGTGCTGGATATCTGCAGAATTCTCAGTGCCGCCGCATCTTCACCTTTCGGGCAGGGCTGC
